# Cytokine Dynamics in Bortezomib‐Induced Peripheral Neuropathy: Challenges in Translating Preclinical Findings to Humans

**DOI:** 10.1111/jns.70090

**Published:** 2026-01-23

**Authors:** Nadine Cebulla, Daniel Schirmer, Eva Runau, Leon Flamm, Calvin Terhorst, Laura Jähnel, Johanna Güse, Nicola Giordani, Annett Wieser, Felicitas Schoch, Marie‐Luise Reinle, Sonja Gommersbach, Aikaterini Papagianni, Xiang Zhou, Hermann Einsele, Ann‐Kristin Reinhold, Heike Rittner, K. Martin Kortüm, Claudia Sommer

**Affiliations:** ^1^ Department of Neurology University Hospital Würzburg Germany; ^2^ Department of Internal Medicine II University Hospital Würzburg Germany; ^3^ Centre for Interdisciplinary Pain Medicine, Department of Anesthesiology, Intensive Care, Emergency and Pain Medicine University Hospital Würzburg Germany

**Keywords:** bortezomib, bortezomib‐induced peripheral neuropathy, chemokines, CIPN, cytokines

## Abstract

**Background and Aims:**

Bortezomib‐induced peripheral neuropathy (BIPN) remains a common treatment side effect in patients with multiple myeloma (MM). Data from rodent models indicate a role of proinflammatory cytokines in BIPN pathophysiology, making them potential therapeutic targets. We therefore tested cytokine levels throughout the course of BIPN in a cohort of MM patients.

**Methods:**

We performed an interim analysis of a monocentric, non‐randomized, observational study including 113 patients with MM. Three groups of patients—within their first cycle of BTZ treatment (FC), with ongoing BTZ treatment at the time of recruiting (OT), and with BTZ treatment in the past (PT)—were compared to controls. Sixteen FC patients were followed up for a median of 6 months. Serum TNF‐α, IL‐6, and CCL2, the cytokines most often implied in the animal models, were analyzed via the ELLA device.

**Results:**

CCL2 levels were not different among our patient groups or in comparison with healthy controls. Compared to healthy controls, the FC group had the highest IL‐6 levels, followed by the PT and then the OT group. The FC group also had higher TNF‐α levels compared to all other groups. Six months after inclusion, patients showed a decrease in TNF‐α levels compared to their baseline. There was no correlation between TNF‐α levels and neuropathy severity or impairment in daily life.

**Interpretation:**

Factors related to MM may influence systemic cytokine levels in BIPN patients, limiting conclusions on their role in BIPN pathophysiology and their utility as drug targets.

## 1 Background

Multiple myeloma (MM) is a plasma cell neoplasm characterized by uncontrolled production of non‐functional antibodies and light chains. MM can lead to severe damage to various organs [1]. Patients suffering from MM are commonly treated with proteasome inhibitors, which exert anti‐MM effects by inhibiting the catalytic activity of proteasomes. Bortezomib (BTZ) is the first‐in‐class proteasome inhibitor and has demonstrated remarkable clinical efficacy in MM [2]. BTZ targets the chymotrypsin‐like site of the 20S proteolytic core within the 26S proteasome, a key component of the ubiquitin‐proteasome pathway. This pathway is required for transcriptional regulation, and nuclear factor‐κB (NF‐κB) is a key transcription factor [3, 4]. BTZ can be administered intravenously or subcutaneously, with subcutaneous application offering a better safety profile without compromising efficacy [5]. In clinical practice, BTZ is often combined with CD38 antibodies (daratumumab/isatuximab), immunomodulatory drugs (thalidomide, lenalidomide, or pomalidomide), and corticosteroids (prednisolone or dexamethasone) [6, 7]. One of the significant side effects of BTZ treatment is BTZ‐induced peripheral neuropathy (BIPN). BIPN is a length‐dependent sensory axonopathy with numbness, tingling, pain, and paresthesias in a symmetrical stocking and glove distribution [8]. Patients affected by BIPN have various sensory deficits (heat and warmth thresholds, touch, and sharpness detection), and in severe cases also pareses [9]. Symptoms may improve after the end of the treatment, but BIPN is still a dose‐limiting complication which leads to dose reduction or even discontinuation of treatment in many cases [10].

The underlying mechanisms that trigger BIPN are poorly understood, and most of the current knowledge on BIPN pathophysiology is derived from rodent studies. Neuroinflammation and inflammatory signaling are thought to play an important role in the development of BIPN. In animal models, BTZ has been shown to induce macrophage infiltration into the sciatic nerve, dorsal root ganglion (DRG), and spinal cord, caused by upregulation of chemokines [11]. Notably, administration of intravenous immunoglobulins reduced macrophage infiltration, heat, and mechanical allodynia in BTZ‐treated rats [12].

BTZ has also been shown to increase the mRNA levels of tumor necrosis factor‐α (TNF‐α) and interleukin‐6 (IL‐6) in the DRG of BTZ‐treated mice, proposing a chronic increased expression of the proinflammatory cytokines TNF‐α and IL‐6 in DRG neurons [13]. Both cytokines can activate the c‐Jun N‐terminal kinase (JNK) pathway [14]. When the TNF‐α synthesis inhibitor thalidomide was administered intraperitoneally, the activation of JNK in the DRG induced by BTZ was significantly blocked. Furthermore, knockout of the TNF‐α receptor TNFR1 decreased mechanical allodynia in BTZ‐treated rats [15]. Similarly, CC‐chemokine ligand 2 (CCL2) expression was increased in the DRG of BTZ‐treated rats [16].

The few studies in humans did not show a consistent trend in the role and behavior of cytokines and chemokines in humans. One study involving 35 MM patients found that individuals who developed BIPN after five cycles of BTZ + dexamethasone treatment had elevated TNF‐α serum levels [17]. In another study, where the authors compared untreated MM patients and treated MM patients with/without neuropathy, the untreated group had the highest TNF‐α plasma levels. The same trend was visible for the IL‐6 levels, with the greatest variation in the untreated group. The majority of these patients were treated with BTZ + dexamethasone + thalidomide [18].

BTZ administration in a mouse model activated the NF‐κB signaling pathway and the inhibition of this pathway attenuated BIPN [13, 19]. In contrast, in cancer cells, BTZ downregulates the NF‐κB dependent induction of IL‐6 in bone marrow stromal cells (BMSCs) triggered by MM cell binding and revokes TNF‐α‐induced upregulation of adhesion molecules on MM cells and BMSCs and also inhibits MM cell to BMSC binding [20].

To date, there is insufficient evidence supporting the translational relevance of the cytokine‐related hypotheses from rodent and cellular models in BIPN, the interpretation of which is even more difficult with the different results of the human studies. The aim of this study was to assess systemic levels of the proinflammatory cytokines TNF‐α and IL‐6 as well as the chemokine CCL2 in a cohort of MM patients and to relate this to the severity of neuropathy in our cohort, to contribute to closing this translational gap.

## 2 Patients and Methods

### 2.1 Study Design and Recruitment

Patients were recruited at the Multiple Myeloma Center of the Department of Internal Medicine II at the University Hospital Würzburg. Here we present an interim analysis of 113 patients from a monocentric, non‐randomized, observational clinical trial (DRKS00025422). All patients were diagnosed with MM according to current International Myeloma Working Group (IMWG) criteria [21] and were included from June 2021 until August 2024. Inclusion criteria were a minimum age of 18 years and the confirmed diagnosis of MM. Exclusion criteria were serious other medical conditions that would prevent the subject from study participation. Patients were divided into three groups: (1) patients in the first cycle of BTZ treatment at the time of recruitment (FC), (2) patients with ongoing treatment with BTZ at the time of recruitment (OT), and (3) patients with BTZ treatment in the past (PT). We followed up 16 FC patients in a median time period of six months after inclusion (range: 4–10 months).

The Ethics Committee of the Medical Faculty of the University of Würzburg approved the study (# 98/20). After oral and written information, discussion, and a reflection period of at least 24 h, patients who agreed to participate signed the consent form and were thus included in the study. Controls for cytokine and chemokine detection in the serum were healthy volunteers without pain or other known neurological conditions (approval by the Ethics Committee of the Medical Faculty of the University of Würzburg # 242/17).

### 2.2 Clinical Assessment

Clinical assessment was performed as previously described [22]. Briefly, patients underwent a standardized neurological examination at the Department of Neurology at the University Hospital Würzburg, including a detailed sensory examination, muscle strength evaluation according to the Medical Research Council (MRC) scale, assessment of the Overall Disability Sum Score (ODSS) and the modified Toronto Clinical Neuropathy Score (mTCNS), quantitative sensory testing (QST), and nerve conduction studies. The mTCNS is derived from the original TCNS and includes a categorical scale of sensory tests [23]. Due to the elimination of reflex tests, the scale reliability was improved, and the mTCNS was more sensitive than the TCNS [24]. Data on previous diagnostic and treatment regimens prior to the admission at our center were extracted from the patients' records.

### 2.3 Grading of Neuropathy

We established a summary report form for our study patients. The report form includes 4 parameters (sensory and motor function, QST, nerve conduction studies) which were defined as abnormal or normal after assessment of the findings by experienced neurologists. The criteria for abnormal findings were defined as previously described [22]. Neuropathy was diagnosed if the pathological findings could be classified as severity grade ≥ 1. Each neuropathy was classified according to the Velcade SmPC [25] shown in Table 1.

**TABLE 1:** Classification of severity grades.

Grade^a^	Criteria
Severity Grade 1	Asymptomatic; loss of deep tendon reflexes or paresthesia
Severity Grade 1 with pain	
Severity Grade 2	Moderate symptoms; limiting instrumental Activities of Daily Living (ADL)^b^
Severity Grade 2 with pain	
Severity Grade 3	Severe symptoms; limiting self‐care ADL^c^
Severity Grade 4	Life‐threatening consequence; urgent intervention indicated and/or severe autonomic neuropathy

^a^ Based on posology modifications in Phase II and III multiple myeloma studies and post‐marketing experience. Grading based on NCI Common Toxicity Criteria (CTCAE) v 4.0.

^b^ Instrumental ADL: refers to preparing meals, shopping for groceries or clothes, using the telephone, managing money, etc.

^c^ Self‐care ADL: refers to bathing, dressing and undressing, self‐feeding, using the toilet, taking medications, and not being confined to bed.

### 2.4 Measurement of CCL2, IL‐6 and TNF‐α in Serum

Venous blood was drawn in the morning and allowed to stand for 30 min. Then it was centrifuged at 1200×*g* for 10 min. The serum was stored at −20°C and thawed immediately before use. Cytokine and chemokine levels were measured using the ELLA device (ProteinSimple, CA, USA), a next generation enzyme‐linked immunosorbent assay (ELISA), according to the manufacturer's instructions by a blinded investigator. The quantification range was 1.52–5780 pg/mL for CCL2, 0.28–2652 pg/mL for IL‐6, and 0.3–1160 pg/mL for TNF‐α. For IL‐6, 2 values had to be excluded from the analysis because they were below the detectable range.

### 2.5 Statistical Analysis

Statistical analyses were performed by using IBM SPSS Statistics Version 29.0 (Armonk, New York, USA) and Prism Version 10.1.2 (GraphPad Software, San Diego, CA). Graphs were created with Prism. Normality of the data was tested by the Shapiro–Wilk normality test. Statistical significance was calculated using an unpaired Kruskal‐Wallis test with Dunn's correction or Fisher's exact test for group comparisons. The Wilcoxon signed rank test was used for the comparison of repeated measures. Statistical significance was set at *p* < 0.05. For seven patients, the MRC sum score; for one patient, the ODSS; for two patients, the motor function; and for one patient, the electrophysiology was not available and therefore not included in the analysis. This analysis includes the clinical data of 70 patients at baseline and 14 healthy controls reported in our first interim analysis [22]. The current analysis is enlarged by the clinical data of 43 baseline investigations and follow‐up data. The data on cytokines and chemokines have not been reported yet.

## 3 Results

### 3.1 Demographics of the Cohort

Demographic data are displayed in Table 2. Our interim analysis included 113 patients (82 men). The median age in the overall cohort was 66 years (range: 31–82), 61 years (range: 42–76) in the FC group, 64 years (range: 46–81) in the OT group, and 68.5 years (range: 31–82) in the PT group.

**TABLE 2:** Demographic data.

Number of patients	Overall cohort, *N* = 113	First cycle of BTZ treatment (FC), *N* = 25	Ongoing BTZ treatment (OT), *N* = 42	BTZ treatment in the past (PT), *N* = 46	Healthy controls (HC), *N* = 14	*p*
Sex (male/female)	82/31	19/6	29/13	34/12	8/6	n.s.
Age in years, median (range)	66 (31–82)	61 (42–76)	64 (46–81)	68.5 (31–82)	60.5 (25–73)	n.s.
Neuropathy	92 (81%)	13 (52%)	36 (86%)	43 (94%)	—	< 0.001
Severity grades						< 0.001
No neuropathy	21 (19%)	12 (48%)	6 (14%)	3 (7%)	—	
Grade 1	41 (36%)	12 (48%)	15 (36%)	14 (30%)	—	
Grade 1 with pain	17 (15%)	—	7 (17%)	10 (22%)	—	
Grade 2	9 (8%)	1 (4%)	5 (12%)	3 (7%)	—	
Grade 2 with pain	22 (19%)	—	8 (19%)	14 (30%)	—	
Grade 3	3 (3%)	—	1 (2%)	2 (4%)	—	
Abnormal findings						
Sensory function	82 (73%)	7 (28%)	34 (81%)	41 (89%)	—	< 0.001
Motor function	33 (29%)	3 (12%)	12 (29%)	18 (39%)	—	n.s.
Nerve conduction studies	72 (64%)	11 (44%)	24 (57%)	37 (80%)	—	< 0.01
QST^a^	76 (67%)	10 (40%)	29 (69%)	37 (80%)	—	< 0.01
ODSS,^b^ median (range)	1 (0–7)	0 (0–5)	1 (0–7)	2 (0–6)	—	n.s.
MRC sum score,^c^ median (range)	120 (99–120)	120 (110–120)	120 (102–120)	120 (99–120)	—	n.s.
mTCNS sum score,^d^ median (range)	9 (0–29)	3 (0–13)	9 (0–23)	12 (0–29)	—	< 0.001
Approximate cumulative dose (mg/m^2^), median (range)	16.6 (0–227.6)	2.6 (0–5.2)	26.65 (3.9–153.4)	19.6 (3.9–227.6)	—	< 0.001
Approximate time since last BTZ application (months), median (range)	2 (0–171)	0 (—)	0 (0–5)	19 (1–171)	—	< 0.001

^a^ Quantitative sensory testing.

^b^ Overall disability sum score.

^c^ Medical research council.

^d^ Modified Toronto clinical neuropathy score.

### 3.2 Severity and Frequency of Neuropathy

**FIGURE 1:**
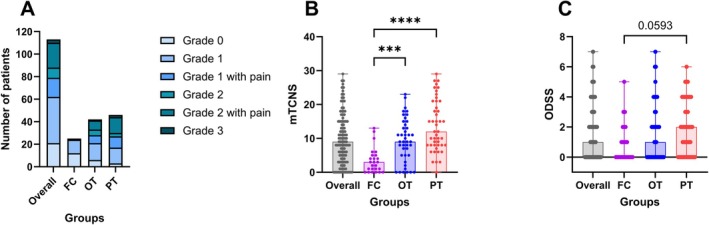
Severity of the neuropathy with grading and scores. (A) Distribution of severity grades; (B) distribution of the mTCNS: overall: *N* = 113; FC: *N* = 25; OT: *N* = 42; PT: *N* = 46; (C) distribution of the ODSS: median ODSS with range: overall: *N* = 112; FC: *N* = 24; OT: *N* = 42; PT: *N* = 46; significance levels: ***p < 0.001, ****p < 0.0001; patients in the first cycle of BTZ treatment at the time of recruitment (FC), patients with ongoing treatment with BTZ at the time of recruitment (OT), patients with BTZ treatment in the past (PT).

Ninety‐two of 113 patients presented with neuropathy, whereby this was mild in the majority, reflected in severity grade 1 (*N* = 41) and severity grade 1 with pain (*N* = 17). Of all groups, the PT group showed a higher frequency of ≥ grade 2 neuropathies (Figure 1A). Most often abnormal were sensory function, nerve conduction studies, and QST. In nerve conduction studies, the majority of the patients showed a typical axonal damage pattern indicated by reduced sural nerve amplitudes, followed by a reduced compound muscle action potential of the tibial nerve without a reduction in nerve conduction velocity.

The OT (*p* = 0.0008) and the PT (*p* < 0.0001) group had higher mTCNS levels compared to the FC group (Figure 1B). In the scoring of the ODSS, the PT group showed a trend towards higher levels compared to the FC group (*p* = 0.059; Figure 1C). The FC and the OT group were currently in a treatment plan, which included BTZ.

### 3.3 CCL2, IL‐6 and TNF‐α in Serum at Time Point of Inclusion

**FIGURE 2:**
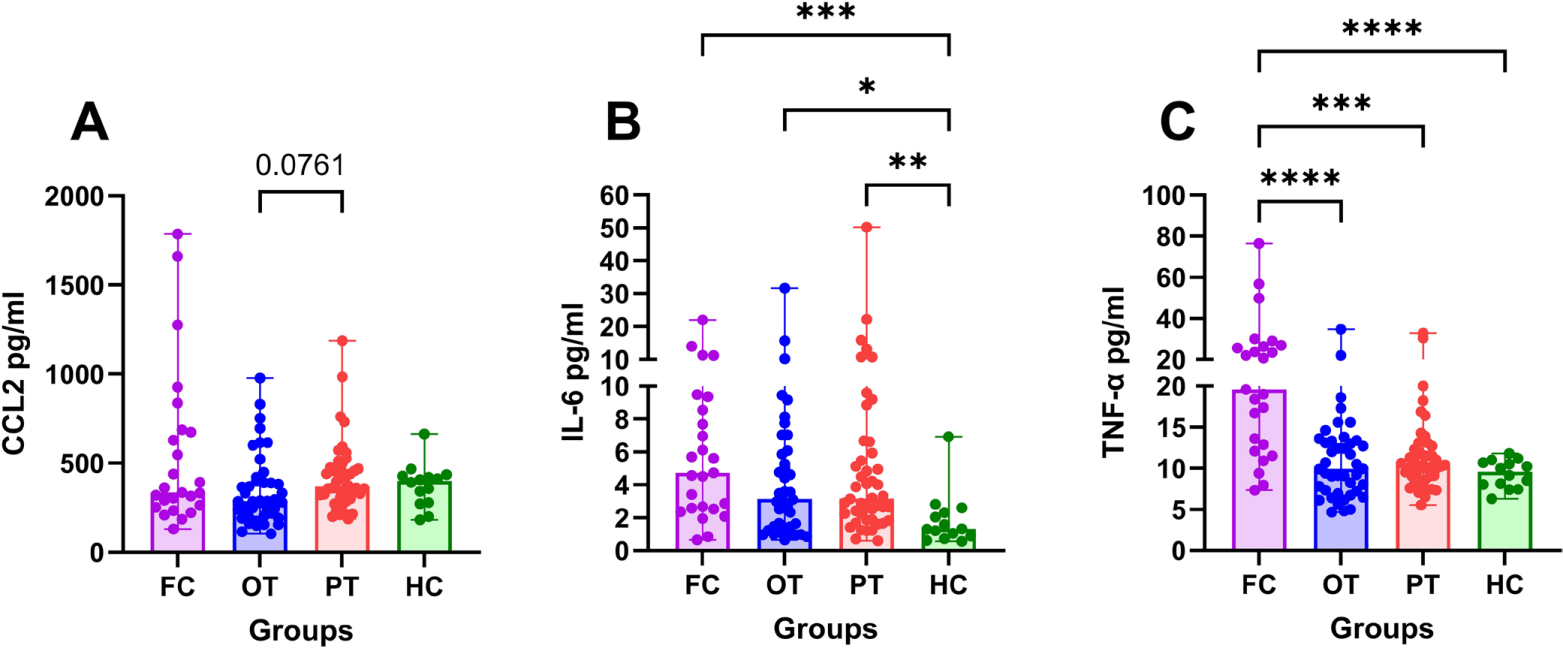
Serum cytokine and chemokine levels in MM patients. Values of CCL‐2 (A), IL‐6 (B), TNF‐α (C), all shown as scatterplots with medians and ranges, significance levels: *p < 0.05, **p < 0.01, ***p < 0.001, ****p < 0.0001; patients in the first cycle of BTZ treatment at the time of recruitment (FC), patients with ongoing treatment with BTZ at the time of recruitment (OT), patients with BTZ treatment in the past (PT), healthy controls (HC).

CCL2 levels were not different among our patient groups or in comparison with healthy controls (Figure 2A). Compared to healthy controls, the FC group had the highest levels of IL‐6 (*p* = 0.0006), followed by the PT (*p* = 0.0058) and then the OT group (*p* = 0.0355) (Figure 2B). The FC group had higher TNF‐α levels compared to all other groups (FC vs. OT: *p* < 0.0001; FC vs. PT: *p* = 0.0002; FC vs. HC: *p* < 0.0001) (Figure 2C). The exact values can be viewed in Table S1.

### 3.4 Longitudinal Data

#### 3.4.1 Frequency and Severity of Neuropathy

To answer the question of whether cytokine and chemokine levels change in the first 6 months after the start of treatment, the period in which neuropathy normally occurs or worsens, we examined 16 patients from the FC group again after a median period of six months (range: 4–10 months) = timepoint 1 (TP1). Nine patients of the original 25 were lost to follow‐up due to deteriorated health conditions (other than neurotoxicity) or treatment complications.

Ten patients already showed signs of neuropathy during the first cycle of BTZ. Six of them were BTZ associated, four were pre‐existing. Reasons for pre‐existing neuropathy were diabetes (2) and pre‐diabetes (2). These patients were mildly affected initially (severity grade 1), except for one patient with severity grade 2. After a median time period of six months, 13 patients were diagnosed with a neuropathy, severity grades had increased, and eight patients had developed pain. Table 3.

**TABLE 3:** Demographic data at timepoint 0 (TP0) and timepoint 1 (TP1).

Number of patients	Timepoint 0 (TP0), *N* = 16	Timepoint 1 (TP1), *N* = 16
Sex, male/female	12/4	
Neuropathy	10 (62.50%)	13 (81.25%)
Severity grades		
No neuropathy	6 (37.50%)	3 (18.75%)
Grade 1	9 (56.25%)	2 (12.50%)
Grade 1 with pain	—	2 (12.50%)
Grade 2	1 (6.25%)	3 (18.75%)
Grade 2 with pain	—	6 (37.50%)
Grade 3	—	—
Abnormal findings		
Sensory function	6 (3%)	11 (69%)
Motor function	2 (13%)	1 (6%)
Nerve conduction studies	7 (44%)	9 (56%)
QST^a^	7 (44%)	11 (69%)
ODSS,^b^ median (range)	0.5 (0–5)	0.5 (0–3)
MRC sum score,^c^ median (range)	120 (110–120)	120 (96–120)
mTCNS sum score,^d^ median (range)	3.5 (0–13)	7 (0–24)

^a^ Quantitative sensory testing.

^b^ Overall disability sum score.

^c^ Medical research council.

^d^ Modified Toronto clinical neuropathy score.

#### 3.4.2 Cytokines and Chemokines

**FIGURE 3:**
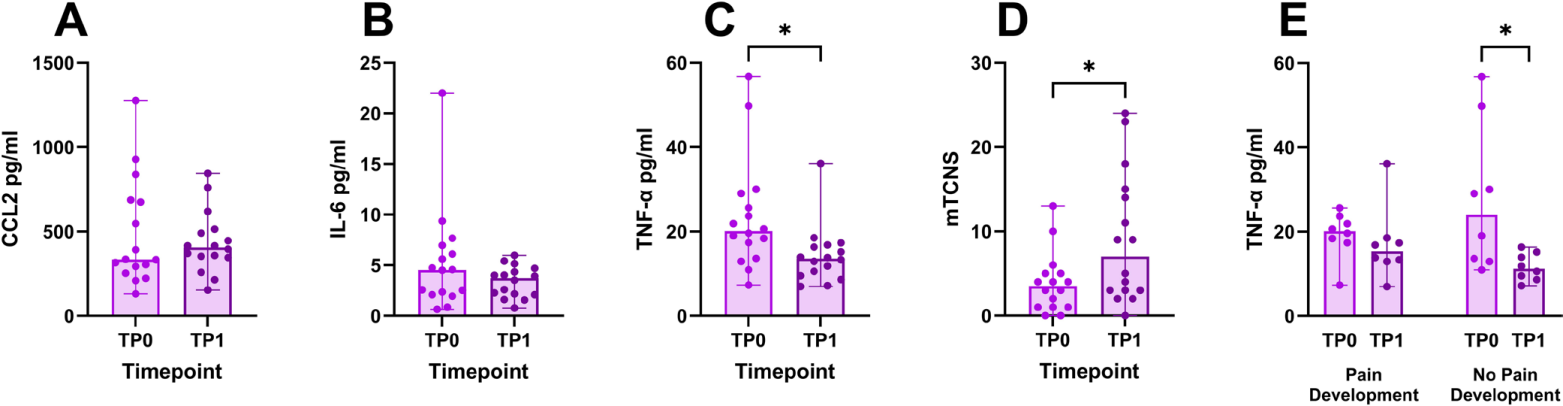
Cytokines, chemokines, and mTCNS at the timepoint of inclusion and at follow‐up. Values of CCL‐2 (A), IL‐6 (B), TNF‐α (C), and the mTCNS (D), all shown as scatterplots with medians and ranges, at TP0 and TP1. (E) TNF‐α is lower at TP1 only in the subgroup of patients without pain development. TP0 = timepoint of inclusion, TP1 = follow‐up after a median time period of 6 months; significance levels: *p < 0.05.

CCL2 and IL‐6 did not show relevant changes over time (Figure 3A,B). TNF‐α decreased at timepoint 1 (TP1) compared to the timepoint of inclusion (TP0; *p* = 0.0182, Figure 3C), whereas mTCNS increased at TP1 (*p* = 0.0127, Figure 3D). The decrease in TNF‐α was driven by the patients without pain development at TP1 (*p* = 0.0234) (Figure 3E). The exact values can be viewed in Table S2.

## 4 Discussion

In this cross‐sectional interim analysis, we found that more BTZ‐treated patients are affected by neuropathy than previously described [7, 26]. The majority of the neuropathy was mild, primarily classified as severity grade 1 with and without pain. At these severity grades, neuropathy can be asymptomatic and was only detected through comprehensive neurological assessment, underscoring the importance of a precise examination. Severity grade 2 with and without pain was more frequent in the group of patients with BTZ treatment in the past, indicating potential progression of neuropathy after completion of the BTZ cycles.

Compared to healthy controls, patients in their first cycle of BTZ treatment (FC) had the highest levels of IL‐6, followed by patients with BTZ in the past (PT) and then the patients with ongoing BTZ treatment (OT). Additionally, the FC group had higher TNF‐α levels compared to all other groups. To interpret these findings, we have to consider different confounding factors.

In the animal models, the first peak of cytokine increase measured in the DRG occurred early in the treatment course. For instance, Alé et al. demonstrated that TNFα mRNA levels increased in the DRGs after just two BTZ injections. This was followed by elevated IL‐6 and TNFR1 levels after a two‐week period, indicating the potential for TNF‐α autoinduction at subsequent time points [13]. Therefore, it is not certain when to expect the highest values in human peripheral blood samples.

On the other hand, animal models do not include the cancer and its effect itself. Cancer cells create an environment in which they can survive and continue to grow rapidly. For this, in MM, the malignant plasma cells (PCs) and the cells of the bone marrow (BM) act in a distinctive interplay, including a complex cytokine signaling, which activates downstream pathways, one being the NF‐κB pathway [27]. To promote survival, MM cells expand to create a premalignant niche within the BM mediated among others by TNF‐α and IL‐6 [28]. Both cytokines also foster MM cell growth in the bone microenvironment [29]. In serum of MM patients with active disease, IL‐6 was reported to be elevated already in 1990 [30]. A later study confirmed elevated IL‐6 and TNF‐α serum levels in newly diagnosed and relapsed/refractory MM patients and proposed these as markers for disease progression and their reduction as a sign of treatment response [28]. In this regard, our data are in line with these findings, with highest cytokine levels observed in newly diagnosed patients and a decline of TNF‐α over the first six months post‐treatment. During this period, the mean mTCNS increased, indicating more severe neuropathies irrespective of declining or stable cytokine levels. Our data also partly align with previous findings from 2021. In their study, they investigated among others plasma cytokine and chemokine levels in untreated and treated MM patients with/without neuropathy. The untreated group in their study is comparable to our FC group, even though our patients already had a low dose of treatment. Their treated groups had cumulative doses of 23.68 ± 5.54 mg/m^2^ (PN group) and 30.92 ± 14.65 mg/m^2^ (no PN group) group, which is comparable to our OT group with a median cumulative dose of 26.65 mg/m^2^. In their study, the untreated group had the highest TNF‐α plasma levels, like our FC group. The same trend was visible for the IL‐6 levels, with the greatest variation in the untreated group [18]. Also in this study, there was no correlation between BIPN severity and TNF‐α or IL‐6 plasma levels. The fact that their untreated patients showed the same trend towards the highest TNF‐α and IL‐6 levels as our FC patients adds significance to the potential relevance of the MM itself in regard to the cytokine levels. Another study from 2024 investigated cytokines in patients with MM before lenalidomide therapy at the time of best response and at disease progression to find a biomarker for clinical outcomes. They showed that TNF‐α levels decreased during lenalidomide therapy. IL‐6 levels were low during lenalidomide therapy but increased when disease progression occurred, indicating that cytokine levels were influenced by the therapy and the disease itself [31].

Since it is therefore not possible to say with certainty whether the cytokines are driven more by the underlying disease or by the therapy itself, and no correlations with BIPN severity were visible, it is difficult to draw a causality between the systemic cytokine levels and BIPN in order to use them as therapeutic targets.

While the regulation of serum cytokine levels in MM patients depends on various factors, in the animal models BTZ is the only variable. In rodent studies, BTZ is typically administered as a monotherapy, whereas in clinical settings, BTZ is commonly used in combination regimens. These agents may influence cytokine and chemokine levels, in addition to the effects of MM itself as outlined above. Immunomodulatory drugs (IMiDs) like thalidomide and lenalidomide have an inhibitory effect on TNF‐α, IL‐1β, and IL‐6 production by MM cells, which is one important part of the tumoricidal effect of these drugs [32, 33]. On the other hand, IMiDs cause an increased activation of natural killer (NK) and T‐cells [29]. Dexamethasone in combination with lenalidomide synergistically inhibits the growth of myeloma cells and thus the tumoricidal effect, but appears to counteract the immune‐enhancing effect by inhibiting the costimulatory effect on T and NK cells [34, 35]. One of the effects of BTZ itself is the alteration of NFkB activation [4]. In our study, most patients receiving ongoing BTZ treatment were on CASSIOPEIA [7] or PERSEUS—regimens [6], which include thalidomide or lenalidomide or were on BTZ containing relapse protocols, such as CASTOR [36], OptimisMM [37] or individualized protocols [38]. Additionally, all these regimens contain dexamethasone. Patients in group 3 (BTZ treatment in the past) were either with lenalidomide as a maintenance therapy or were without therapy due to a disease‐free period (details in Table S3). If one now considers that these many different substances can also have an effect on systemic cytokine and chemokine levels, it is even more difficult to find a good rationale to use these cytokines as targets for pharmacological intervention.

Most of the effects in rodent models were observed in DRG, spinal cord, or sciatic nerves. These tissues are inaccessible in human clinical studies, limiting direct comparisons. In order to have a marker in humans that can be easily determined and on which a pharmacological intervention can then be based, the tissue examined should be easy to obtain, just like peripheral blood or potentially skin. In humans, various comorbidities can influence the outcome of diseases. In our study, some patients already had pre‐existing neuropathy, and these patients had more severe neuropathies after 6 months of follow‐up, even though cytokines and chemokines did not differ, possibly due to the small sample size. Rodent BIPN models did not replicate pre‐existing neuropathic conditions, nor did they consider how these conditions might influence cytokine and chemokine responses, as the implementation of all these factors in one model is very difficult. This fundamental difference complicates the direct translation of preclinical findings to human BIPN.

Given all these additional factors influencing cytokine levels in MM patients, it is not surprising that there were no correlations between cytokine levels and BIPN severity.

Our study has some limitations. First of all, we have a cross‐sectional design with a variation in the timepoints of the blood withdrawal between and within the groups. This is caused by the fact that we had to adjust our blood withdrawal to the individual everyday clinical practice of each patient because of the complexity of the disease and the organization of the therapy. The ideal conditions for the comparisons would be a defined timepoint for each group. In the real world, it is hard to manage this because patients are coming to our center at very different timepoints in their disease. The second important point is the missing baseline data from newly diagnosed MM patients and without any treatment and the comparison of these cytokine and chemokine levels with the different groups. Lastly, we only had a longitudinal dataset of 16 patients, even though 25 patients were included in the baseline investigations. Reasons for dropouts were a reduced general health condition due to cancer, infection, isolation due to low immune cells, or a continuation of the therapy plan at a hospital closer to the patient's home.

## 5 Conclusion

Our study highlights the complexity of cytokine and chemokine regulation in MM patients with and without BIPN. The presence of multiple confounding factors, including the disease itself and treatment effects, challenges the rationale for targeting TNF‐α, IL‐6, and CCL2 as therapeutic options for BIPN. The results to date from animal models are based on well‐designed and robust studies, but are difficult to translate into humans. Given the discrepancies between preclinical and clinical observations with regard to the investigated tissue and the treatment regimens, future research should therefore focus on better‐defined animal and human models that incorporate disease‐specific factors and treatment regimens to refine our understanding of BIPN pathophysiology.

## Authors´ Contributions

All authors contributed to the study conception and execution. Patient recruitment, sample preparation, and data collection were performed by C.S., N.C., K.M.K., D.S., E.R, L.F., C.T., L.J., J.G., N.G., A.W., F.S., M.‐L.R., S.G., X.Z., A.P., and A.‐K.R. H.E. and H.R. contributed to study planning and implementation. K.M.K. and C.S. are the principal investigators and designed the study. The data analysis was performed by N.C. The first draft of the manuscript was written by N.C. and C.S. All authors commented on previous versions of the manuscript. All authors read and approved the final manuscript.

## Funding

This study was part of KFO5001 ResolvePAIN, funded by the Deutsche Forschungsgemeinschaft (DFG, German Research Foundation) – Project ID: 426503586.

## Conflicts of Interest

C.S. is Editor of the “European Journal of Neurology” and is President Elect of the Peripheral Nerve Society. H.R. is Section Editor for “Pain Reports”. The authors have no competing interests to declare that are relevant to the content of this article.

## Supporting information


**TABLE S1:** Serum cytokine and chemokine levels in MM patients with BTZ treatment.
**TABLE S2:** Cytokines, chemokines and at timepoint of inclusion and at follow‐up.
**TABLE S3:** Main chemotherapeutic agents administered at the time of investigation.

## Data Availability

The datasets used and/or analyzed during the current study are available from the corresponding author on reasonable request.
