# Supplementary material for: Cytokine Dynamics in Bortezomib‐Induced Peripheral Neuropathy: Challenges in Translating Preclinical Findings to Humans
Source: J Peripher Nerv Syst. 2026 Jan 23;31(1):e70090. doi: 10.1111/jns.70090 (PMC12831098; doi:10.1111/jns.70090)
Supplement: Supplementary file 1 — TABLE S1: Serum cytokine and chemokine levels in MM patients with BTZ treatment. TABLE S2: Cytokines, chemokines and at timepoint of inclusion and at follow‐up. TABLE S3: Main chemotherapeutic agents administered at the time of investigation. [file JNS-31-0-s001.docx]

**Cytokine Dynamics in Bortezomib-Induced Peripheral Neuropathy: Challenges in Translating Preclinical Findings to Humans**

Nadine Cebulla^1^, Daniel Schirmer^1^, Eva Runau^1^, Leon Flamm^1^, Calvin Terhorst^1^, Laura Jähnel^1^, Johanna Güse^1^, Nicola Giordani^1^, Annett Wieser^1^, Felicitas Schoch^1^, Marie-Luise Reinle^1^, Sonja Gommersbach^1^, Aikaterini Papagianni^1^, Xiang Zhou^2^, Hermann Einsele^2^, Ann-Kristin Reinhold^3^, Heike Rittner^3^, K. Martin Kortüm^2^, Claudia Sommer^1,3^
^1^: University Hospital Würzburg, Department of Neurology, 2: University Hospital Würzburg, Department of Internal Medicine II, 3: University Hospital Würzburg, Centre for Interdisciplinary Pain Medicine, Department of Anesthesiology, Intensive care, Emergency and Pain Medicine

Supplementary Information

S1: Serum cytokine and chemokine levels in MM patients with BTZ treatment

|  | **FC** | **N** | **OT** | **N** | **PT** | **N** | **HC** | **N** |
| --- | --- | --- | --- | --- | --- | --- | --- | --- |
| **CCL2**  Median  Range | 336 pg/ml  (131-1785 pg/ml) | 25 | 287 pg/ml  (104-977 pg/ml) | 42 | 369 pg/ml  (187-1187 pg/ml) | 46 | 400 pg/ml  (183-664 pg/ml) | 14 |
| **IL-6**  Median  Range | 4.73 pg/ml  (0.65-22 pg/ml) | 25 | 3.15 pg/ml  (0.66-31.7 pg/ml) | 40 | 3.17 pg/ml  (0.61-50.2 pg/ml) | 46 | 1.32 pg/ml;  (0.56-6.93) | 14 |
| **TNF-α**  Median  Range | 19.6 pg/ml  (7.34-76.4 pg/ml) | 25 | 9.91 pg/ml  (4.66-34.6 pg/ml) | 42 | 10.4 pg/ml  (5.53-32.8 pg/ml) | 46 | 9.57 pg/ml  (6.3-11.8 pg/ml) | 14 |

S2: Cytokines, chemokines and at timepoint of inclusion and at follow-up

|  | **TP0** | **N** | **TP1** | **N** |
| --- | --- | --- | --- | --- |
| **CCL2**  Median  Range | 334.5  (131-1275) | 16 | 405  (153-844) | 16 |
| **IL-6**  Median  Range | 4.54  (0.65-22) | 16 | 3,73  (0.79-5.99) | 16 |
| **TNF-α**  Median  Range | 20.1  (7.34-56.7 | 16 | 13.55  (6.96-36.1) | 16 |

|  | **Pain Development** | | **N** | | **No Pain Development** | | **N** | |
| --- | --- | --- | --- | --- | --- | --- | --- | --- |
|  | **TP0** | **TP1** |  | | **TP0** | **TP1** |  | |
| **TNF-α**  Median  Range | 20.1  (7.34-25.6) | 15.3  (6.96-36.1) | 8 | 8 | 24.0  (10.90-56.7) | 11.2  (7.15-16.3) | 8 | 8 |

S3: main chemotherapeutic agents administered at the time of investigation

| **Patient** |  | **Patient** |  |
| --- | --- | --- | --- |
| **1** | Daratumumab, Pomalidomide, Dexamethasone | **58** | Daratumumab, Carfilzomib, Cyclophosphamide, Dexamethasone |
| **2** | Daratumumab, Lenalidomide, Dexamethasone | **59** | - |
| **3** | Daratumumab, Pomalidomide, Dexamethasone | **60** | - |
| **4** | Cyclophosphamide, Doxorubicin, Etoposide, Cisplatin, Thalidomide, Dexamethasone, | **61** | Lenalidomide |
| **5** | Carfilzomib, Cyclophosphamide, Dexamethasone | **62** | Daratumumab, Lenalidomide, Dexamethasone |
| **6** | Belantamab mafodotin, Thalidomide, Dexamethasone | **63** | Dexamethasone |
| **7** | Daratumumab, Carfilzomib, Thalidomide, Cisplatin, Etoposide, Cyclophosphamide, Dexamethasone | **64** | Daratumumab, Thalidomide, Dexamethasone |
| **8** | Daratumumab, Pomalidomide, Dexamethasone | **65** | Daratumumab, Thalidomide, Dexamethasone |
| **9** | Daratumumab, Lenalidomide Dexamethasone | **66** | - |
| **10** | Daratumumab, Lenalidomide Dexamethasone | **67** | Daratumumab, Thalidomide, Dexamethasone |
| **11** | Daratumumab, Pomalidomide, Dexamethasone | **68** | Lenalidomide |
| **12** | - | **69** | - |
| **13** | Cyclophosphamide, Etoposide | **70** | Daratumumab, Lenalidomide, Dexamethasone |
| **14** | Cyclophosphamide, Etoposide | **71** | Daratumumab, Lenalidomide, Dexamethasone |
| **15** | Daratumumab, Pomalidomide, Dexamethasone | **72** | Lenalidomide |
| **16** | Daratumumab, Pomalidomide, Dexamethasone | **73** | - |
| **17** | Daratumumab, Lenalidomide Dexamethasone | **74** | Ixazomib, Lenalidomide, Dexamethasone |
| **18** | Daratumumab, Pomalidomide, Dexamethasone | **75** | - |
| **19** | Daratumumab, Carfilzomib, Lenalidomide, Dexamethasone | **76** | Lenalidomide |
| **20** | Daratumumab, Carfilzomib, Cisplatin, Etoposide, Cyclophosphamide, Dexamethasone, Thalidomide | **77** | - |
| **21** | Melphalan | **78** | Pomalidomide, Dexamethasone |
| **22** | Daratumumab, Lenalidomide Dexamethasone | **79** | Daratumumab, Dexamethasone |
| **23** | Cyclophosphamide, Doxorubicin, Etoposide, Cisplatin, Thalidomide, Dexamethasone | **80** | Daratumumab, Dexamethasone |
| **24** | Cyclophosphamide, Doxorubicin, Etoposide, Cisplatin, Thalidomide, Dexamethasone | **81** | Lenalidomide, Dexamethasone |
| **25** | Daratumumab, Pomalidomide, Dexamethasone | **82** | Daratumumab, Lenalidomide, Dexamethasone |
| **26** | Daratumumab, Lenalidomide Dexamethasone | **83** | Daratumumab, Lenalidomide, Dexamethasone |
| **27** | Daratumumab, Pomalidomide, Dexamethasone | **84** | - |
| **28** | - | **85** | Daratumumab, Thalidomide, Dexamethasone |
| **29** | Daratumumab, Lenalidomide, Dexamethasone | **86** | Daratumumab, Thalidomide, Dexamethasone |
| **30** | Daratumumab, Lenalidomide, Dexamethasone | **87** | Daratumumab, Thalidomide, Dexamethasone |
| **31** | Lenalidomide | **88** | Daratumumab, Thalidomide, Dexamethasone |
| **32** | - | **89** | Daratumumab, Lenalidomide, Dexamethasone |
| **33** | Belantamab mafododitin, Dexamethasone | **90** | Ixazomib, Lenalidomide, Dexamethasone |
| **34** | Lenalidomide | **91** | - |
| **35** | - | **92** | Daratumumab, Lenalidomide, Dexamethasone |
| **36** | - | **93** | Daratumumab, Lenalidomide, Dexamethasone |
| **37** | Lenalidomide | **94** | Daratumumab, Lenalidomide, Dexamethasone |
| **38** | Daratumumab, Pomalidomide, Dexamethasone | **95** | Daratumumab, Thalidomide, Dexamethasone |
| **39** | Daratumumab, Thalidomide, Dexamethasone | **96** | - |
| **40** | Ifosfamide, Uromitexane, Etoposide, Epirubicine | **97** | Teclistamab |
| **41** | Daratumumab, Pomalidomide, Dexamethasone | **98** | - |
| **42** | Carfilzomib, Cyclophosphamide, Dexamethasone | **99** | Daratumumab, Thalidomide, Dexamethasone |
| **43** | Daratumumab, Lenalidomide, Cyclophosphamide, Dexamethasone | **100** | Daratumumab, Lenalidomide, Dexamethasone |
| **44** | Daratumumab, Lenalidomide Dexamethasone | **101** | Isatuximab, Lenalidomide, Dexamethasone |
| **45** | Melphalan | **102** | Isatuximab, Lenalidomide, Dexamethasone |
| **46** | Daratumumab, Dexamethasone | **103** | Daratumumab, Thalidomide, Dexamethasone |
| **47** | Daratumumab, Pomalidomide, Dexamethasone | **104** | Isatuximab, Lenalidomide, Dexamethasone |
| **48** | - | **105** | Isatuximab, Lenalidomide, Dexamethasone |
| **49** | Daratumumab, Thalidomide, Dexamethasone | **106** | Teclistamab, Lenalidomide, Daratumumab |
| **50** | Daratumumab, Lenalidomide, Dexamethasone | **107** | Daratumumab, Thalidomide, Dexamethasone |
| **51** | Daratumumab, Pomalidomide, Dexamethasone | **108** | Daratumumab, Thalidomide, Dexamethasone |
| **52** | Daratumumab, Carfilzomib, Dexamethasone | **109** | Teclistamab, Lenalidomide, Dexamethasone |
| **53** | Melphalan | **110** | Daratumumab, Lenalidomide, Dexamethasone |
| **54** | Melphalan | **111** | Daratumumab, Lenalidomide, Dexamethasone |
| **55** | Daratumumab, Pomalidomide, Dexamethasone | **112** | Daratumumab, Lenalidomide, Dexamethasone |
| **56** | Daratumumab, Thalidomide, Dexamethasone | **113** | Daratumumab, Lenalidomide, Dexamethasone |
| **57** | Daratumumab |  | |
